# The consequences of host heterogeneity for parasite transmission depend on how host infectiousness is determined

**DOI:** 10.1111/1365-2656.70329

**Published:** 2026-07-31

**Authors:** Jacob A. Cohen, Andrew D. Dean, Mark Viney, Andy Fenton

**Affiliations:** ^1^ Department of Evolution, Ecology and Behaviour Institute of Infection, Veterinary and Ecological Sciences, University of Liverpool Liverpool UK; ^2^ Present address: Department of Biology University of Oxford Oxford UK

**Keywords:** disease ecology, epidemiological model, host heterogeneity, infectiousness, parasite transmission, susceptibility

## Abstract

1. Host heterogeneities in susceptibility and infectiousness can affect parasite transmission, but standard analyses typically consider a limited range of epidemiological scenarios.
2. We propose two contrasting phenomenological scenarios that capture a wide range of processes by which host infectiousness is determined and explore how these scenarios affect the impact of host heterogeneities on parasite transmission.
3. Specifically, we contrast two limiting cases for how host infectiousness may be determined: ‘Recipient dependence’ (RD), where a host's infectiousness is a fixed characteristic of the recipient host being infected, and ‘Donor dependence’ (DD), where a host's infectiousness is determined at the time of infection by properties of the donor host that infected them.
4. Contrasting susceptible‐infected models of these two phenomenological scenarios show that under RD, *R*_0_ is driven by population‐level covariance between susceptibility and infectiousness, but for DD scenarios, it is driven by the maximum infectiousness present in the population.
5. Our results show that the RD and DD scenarios generate distinctive epidemiological signatures. Although many host–parasite systems are likely to lie somewhere along a RD–DD continuum, with infectiousness influenced by both characteristics of the recipient and donor host, understanding these limiting cases provides a valuable framework for interpreting how host heterogeneity influences transmission in more complex systems.

## Graphical abstract

This study proposes two contrasting scenarios for how host infectiousness is determined: recipient dependence and donor dependence. These scenarios fundamentally alter how host heterogeneity shapes parasite transmission, demonstrating that the process by which infectiousness is determined is crucial for understanding the consequences of host heterogeneity.

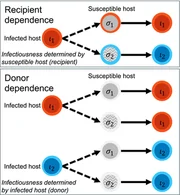

## 1 INTRODUCTION

Individuals can vary substantially in their propensities to be infected by and transmit parasites (VanderWaal & Ezenwa, 2016). These individual‐level host heterogeneities can have significant effects on the transmission of parasites and so affect patterns of infection in host populations. Superspreaders—hosts that are responsible for a disproportionate number of transmission events—are a well‐known, if extreme, example of such host heterogeneity (Lemieux et al., 2021; Lloyd‐Smith et al., 2005; Woolhouse et al., 1997). Despite the commonly accepted importance of host heterogeneity, there has been somewhat limited investigation of this, particularly in understanding the epidemiological consequences of how a host's infectiousness is determined.

Parasite transmission is a function of three factors: (i) host susceptibility, which is the host's propensity to become infected following parasite exposure, (ii) host infectiousness, the capacity of an infected host to transmit parasites and (iii) host contact rate, the rate of transmission‐relevant contacts, dependent on the transmission mode of the parasite in question (McCallum et al., 2017). Variation among individual hosts in any of these traits can alter parasite transmission dynamics in a host population (Barlow, 2000; Coutinho et al., 1999; Dwyer et al., 1997; Kong et al., 2016; Matthews et al., 2006; Stephenson et al., 2017; Streicker et al., 2013; White et al., 2018). Importantly, heterogeneities in these three traits can exist simultaneously (so‐called ‘coupled heterogeneities’), and potentially interact with each other, altering transmission dynamics (Hickson & Roberts, 2014; Miller, 2007; Vazquez‐Prokopec et al., 2016; Yates et al., 2006). For instance, it has been shown that covariation between host heterogeneities can increase or decrease the parasite's basic reproduction number, *R*_0_, depending on the traits considered and whether the covariation is positive or negative (Lloyd et al., 2020; Vazquez‐Prokopec et al., 2016).

To date, most studies investigating the consequences of host heterogeneities on parasite transmission dynamics implicitly assume that a host's infectiousness is a fixed characteristic of the ‘recipient’ individual being infected (Coutinho et al., 1999; Lloyd et al., 2020; Miller, 2007; Yates et al., 2006). We call this ‘Recipient dependence’ (RD) because the infectiousness of the newly infected host (the recipient) is solely determined by characteristics of that individual. There are a range of processes which may lead to RD determination of host infectiousness. For example, hosts might differ in terms of genotype (Anacleto et al., 2019), nutritional status (Cornet et al., 2014), behavioural phenotype (Araujo et al., 2016) or social connectedness (Adelman et al., 2015; Mantzaris & Higham, 2013), all of which result in levels of infectiousness linked to those characteristics of the recipient host. Generally, most studies of host heterogeneities implicitly assume RD‐based scenarios (e.g. Coutinho et al., 1999; Lloyd et al., 2020; Miller, 2007; Yates et al., 2006). In such systems, heterogeneity can either increase or decrease *R*_0_, depending on whether the covariance between host susceptibility and infectiousness is positive or negative (Lloyd et al., 2020).

At the opposite end of this conceptual spectrum are scenarios where the infectiousness of a newly infected host depends on characteristics of the ‘donor’ host that infected it. We call this ‘Donor dependence’ (DD) because the infectiousness of the newly infected host is determined by the infecting host (the donor). DD may occur, for example, when the parasite load received by a newly infected host determines how infectious that host becomes (Strong et al., 2015), such as when highly infectious hosts (i.e. superspreaders) generate other highly infectious hosts (Beldomenico, 2020; Wanelik et al., 2023). It can also capture situations where co‐circulating parasite strains differ in their infectivity (Brault et al., 2004; Dai et al., 2013; Medina et al., 2018), so that hosts infected with highly infective parasite strains then become highly infectious hosts themselves. Critically, and in contrast to the RD scenario, in the DD limiting case, a host's infectiousness is determined solely by the identity of the donor host that infected them. While superspreaders may provide an intuitive example of DD processes, most theoretical treatments of host heterogeneity implicitly assume that infectiousness is an intrinsic characteristic of infected hosts and therefore align more closely with RD (Fujie & Odagaki, 2007; Kuylen et al., 2022; Lau et al., 2017; Lloyd‐Smith et al., 2005). Consequently, alternative mechanisms in which infectiousness is determined by the donor host have received comparatively little explicit attention.

Importantly, the RD and DD scenarios are not distinguished by whether transmission (the probability of infection given contact) depends on recipient or donor traits; in both RD and DD, the probability of infection is modelled as the product of recipient susceptibility and donor infectiousness. Instead, we distinguish RD and DD by how the infectiousness of a newly infected host is determined (Figure 1). Under RD, infectiousness is an intrinsic property of the recipient host that is fixed prior to infection. Under DD, infectiousness is undetermined in an (uninfected) recipient host and, upon infection, is acquired from the donor host responsible for transmission. These distinct mechanisms by which a host's infectiousness is determined generate different population‐level structures of infectiousness, and therefore lead to contrasting epidemiological outcomes. Thus, we propose that RD and DD represent two limiting cases for conceptualising how a host's infectiousness is determined, distinguished by the identity of the host (the recipient or the donor) that determines the infectiousness of a newly infected host. This distinction underpins the analyses that follow and explains the differences between the RD and DD limiting cases considered here.

**FIGURE 1:**
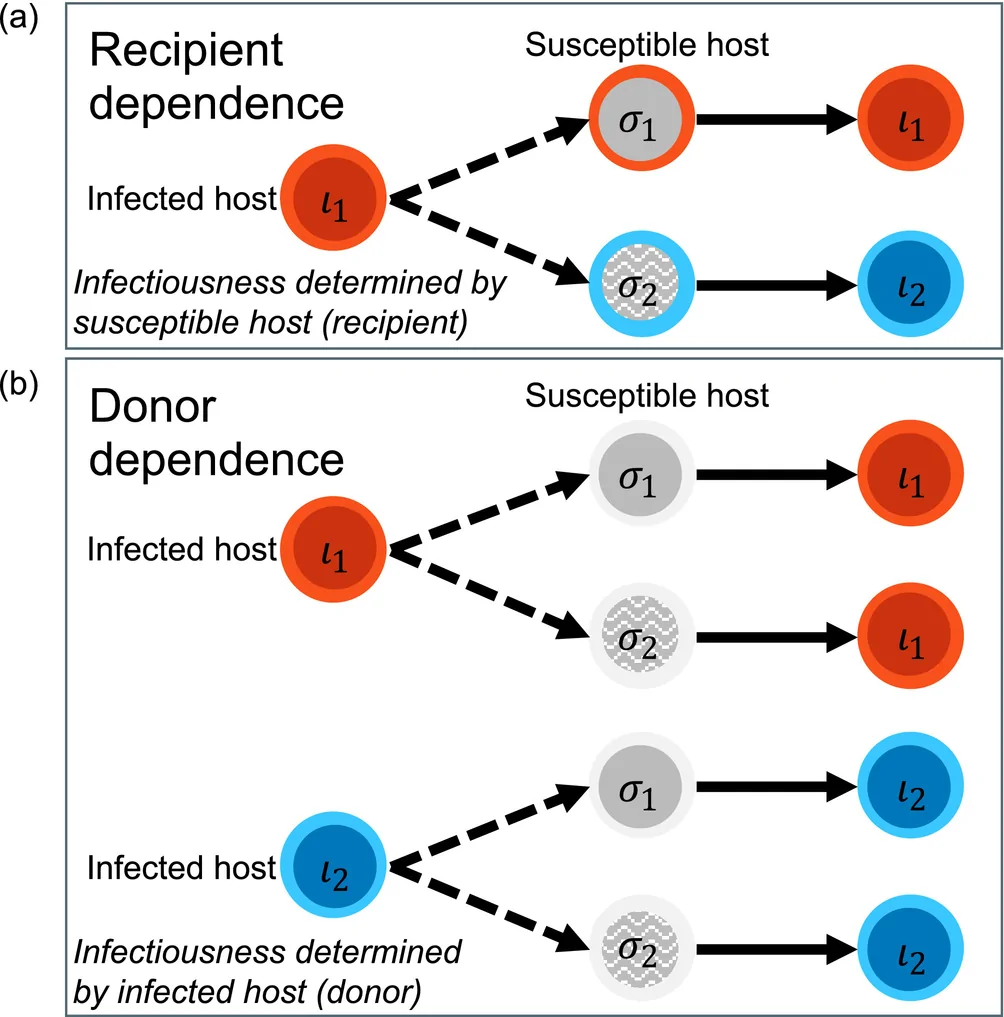
Schematic for the two scenarios for the determination of host infectiousness: recipient dependence (RD) (a) and donor dependence (DD) (b). Each circle represents an individual host. Susceptible hosts have susceptibility traits $\sigma_{1}$ (solid grey circles) or $\sigma_{2}$ (patterned grey circles), which denote two different levels of susceptibility to a parasite. Infected hosts have infectiousness traits $\iota_{1}$ (red) or $\iota_{2}$ (blue), which denote two different levels of infectiousness. The colour of the circle outline denotes the infectiousness trait of an individual. Dashed arrows denote transmission‐relevant contact between two hosts, where the likelihood of transmission is ι×σ. Solid arrows denote a susceptible host becoming infected. Note that susceptible individuals under RD have infectiousness traits (red or blue outlines) while susceptible individuals under DD do not (white outlines). Under RD infectiousness is an intrinsic property of the susceptible host, while under DD infectiousness is a trait inherited from the donor host responsible for transmission.

We note that most host–parasite systems are unlikely to correspond exactly to either pure RD or pure DD. Many real systems are likely to occupy intermediate positions along a RD–DD continuum, with infectiousness shaped by both recipient and donor characteristics. However, the balance of those processes may often be dominated by one scenario or the other. As such, we view RD and DD as two limiting cases that capture alternative mechanisms by which infectiousness can be determined. Analysing these limiting cases therefore provides a valuable starting point because it identifies the distinct epidemiological signatures generated by each mechanism and establishes expectations for systems in which both processes operate simultaneously.

We suggest that the differentiation of the RD and DD scenarios will lead to different patterns of host infectiousness in a population and, crucially, alter the impact of host heterogeneities on parasite transmission. Previous work (e.g. Lloyd et al., 2020; Vazquez‐Prokopec et al., 2016; Wanelik et al., 2023) has implicitly used one or the other of these scenarios but has not systematically explored how they differ in affecting the impact of coupled heterogeneities in susceptibility and infectiousness on parasite transmission. To investigate this, we develop Susceptible‐Infected (S‐I) compartmental models that incorporate heterogeneity in susceptibility and infectiousness under both RD and DD scenarios. We then analyse these models to determine how RD and DD affect the relationships between those host heterogeneities, parasite transmission and host population dynamics. Accordingly, we focus mainly on *R*_0_ and equilibrium host abundance, which captures epidemic potential and long‐term population‐level consequences of parasite transmission; we consider other metrics, such as equilibrium infection prevalence in the Supporting Information. We find that how infectiousness is determined—RD versus DD—can have substantial effects on parasite transmission, particularly affecting the primary drivers of the basic reproduction number (*R*_0_).

## 2 MATERIALS AND METHODS

### 2.1 Model framework

To maintain analytical tractability and comparability with prior work on coupled heterogeneities, we adopted a simple S‐I framework without host density regulation or recovery, and assuming density‐dependent transmission. These assumptions allowed us to isolate the consequences of how infectiousness is determined, without introducing additional feedbacks or complexity that would obscure interpretation of the RD and DD distinction. The standard density‐dependent S‐I model in a homogeneous population of size *N* divided into susceptible (S) and infected (I) sub‐populations (Anderson & May, 1991) is given by

(1) $$\frac{\mathrm{dS}}{\mathrm{dt}}=\mathrm{bN}-\mathrm{\beta SI}-\mathrm{dS},$$

(2) $$\begin{array}{c} \frac{\mathrm{dI}}{\mathrm{dt}}=\mathrm{\beta SI}-\left(d+\alpha \right)I, \end{array}$$

where N=S+I, b is the birth rate, d the baseline mortality rate and α the parasite‐induced mortality rate. The transmission coefficient β, while often written as a simple constant, actually incorporates both contact rate (κ) and infection probability given a contact (ν) (Begon et al., 2002), yielding

(3) $$\begin{array}{c} \beta =\kappa \nu . \end{array}$$

The infection probability ν can be further partitioned into the product of recipient susceptibility (σ) and donor infectiousness (ι),

(4) $$\begin{array}{c} \nu =\sigma \iota , \end{array}$$

where σ and ι take values in the interval [0,1], with higher values representing greater susceptibility or greater infectiousness, respectively. Host heterogeneity in susceptibility and infectiousness can then be incorporated by dividing the population into sub‐populations comprising individuals that share the same values of susceptibility and infectiousness. Precisely how this is done depends on whether infectiousness is determined by a RD or DD scenario. Importantly, in both the RD and DD scenarios, infection probability depends on both recipient susceptibility and donor infectiousness (Equations 3 and 4). The distinction between the scenarios does not concern whether transmission depends on recipient or donor traits, but rather on how infectiousness is assigned to hosts following infection.

### 2.2 Recipient dependence

Under this scenario, recipient hosts are assigned to an infected sub‐population based on a fixed, pre‐determined trait inherent to that individual. Supposing that there are n unique pairs of trait values $\left(\sigma_{j},\iota_{j}\right)$, 1≤j≤n, we divide the population into n susceptible sub‐populations $S_{j}$, and n infected sub‐populations $I_{j}$, where the jth sub‐populations share the jth trait pair. Thus, a RD heterogeneous analogue of Equations (1) and (2) is

(5) $$\begin{array}{c} \frac{dS_{j}}{\mathrm{dt}}=\frac{\mathrm{bN}}{n}-\kappa \sigma_{j}S_{j}\sum_{m=1}^{n}\iota_{m}I_{m}-dS_{j}, \end{array}$$

(6) $$\begin{array}{c} \frac{{\mathrm{dI}}_{j}}{\mathrm{dt}}=\kappa \sigma_{j}S_{j}\sum_{m=1}^{n}\iota_{m}I_{m}-\left(d+\alpha \right)I_{j}, \end{array}$$

where we have additionally assumed that birth rate b, baseline mortality rate d and parasite‐induced mortality rate α, are equal across sub‐populations. For the sake of simplicity, births are evenly distributed across susceptible sub‐populations, effectively assuming non‐inherited host heterogeneity, a phenomenon that has been previously observed, for example, in *Daphnia magna* (Ben‐Ami et al., 2008). We emphasise that in the RD scenario, the fixed traits of individuals determine the susceptible and infected sub‐population to which they belong, so that all individuals in a susceptible sub‐population move to the same infected sub‐population upon becoming infected.

### 2.3 Donor dependence

In this scenario, recipient hosts acquire their infectiousness trait when they become infected. Thus, individuals from the same susceptible sub‐population may enter different infected sub‐populations, depending on the infectiousness of the donor host responsible for their infection. As in the RD scenario, we divide the susceptible sub‐population into $n_{S}$ sub‐populations $S_{j}$, each with susceptibility $\sigma_{j}$, $1\leq j\leq n_{S}$. Under the DD scenario, however, individuals are assigned the infectiousness of the donor host that infected them. We therefore define $I_{j,k}$ to be those individuals with susceptibility $\sigma_{j}$ that were infected by an individual with infectiousness $\iota_{k}$, $0\leq k\leq n_{I}$, and so now share that same infectiousness value. Thus, a DD heterogeneous analogue of Equations (1) and (2) is

(7) $$\begin{array}{c} \frac{dS_{j}}{\mathrm{dt}}=\frac{\mathrm{bN}}{n_{S}}-\kappa \sigma_{j}S_{j}\sum_{m=1}^{n_{I}}\sum_{l=1}^{n_{S}}\iota_{m}I_{l,m}-dS_{j}, \end{array}$$

(8) $$\begin{array}{c} \frac{{\mathrm{dI}}_{j,k}}{\mathrm{dt}}=\kappa \sigma_{j}\iota_{k}S_{j}\sum_{l=1}^{n_{S}}I_{l,k}-\left(d+\alpha \right)I_{j,k}. \end{array}$$

b, d and α are again assumed to be equal for all infected sub‐populations. Unlike the RD model, the infected sub‐population that the recipient host will join is not pre‐determined before infection, so individuals in the same susceptible sub‐population do not always move to the same infected sub‐population upon infection. As such, the DD model has $n_{s}$ susceptible sub‐populations and $n_{S}n_{I}$ infectious sub‐populations, whereas the RD model has equal numbers of both susceptible and infected sub‐populations.

Equations (7) and (8), which will be useful when quantifying population‐level heterogeneity, can be simplified by defining $I_{k}=\sum_{j=1}^{n_{S}}I_{j,k}$, that is, the sum of all individuals with the same infectiousness value, regardless of their initial susceptibility value. Summing Equation (8) over $0\leq j\leq n_{S}$ yields

(9) $$\begin{array}{c} \frac{dS_{j}}{\mathrm{dt}}=\frac{\mathrm{bN}}{n_{S}}-\kappa \sigma_{j}S_{j}\sum_{m=1}^{n_{I}}\iota_{m}I_{m}-dS_{j}, \end{array}$$

(10) $$\begin{array}{c} \frac{dI_{k}}{\mathrm{dt}}=\kappa \iota_{k}I_{k}\sum_{m=1}^{n_{S}}\sigma_{m}S_{m}-\left(d+\alpha \right)I_{k}, \end{array}$$

describing the dynamics of all infected individuals with infectiousness $\iota_{k}$. This simplified form of the system is useful for calculating *R*_0_. Note that we have used the same notation in the RD and the DD models to define similar but not precisely equivalent variables, and therefore we rely on context to provide clarity about which is being referred to throughout the remainder of this work.

### 2.4 Quantifying population‐level heterogeneity

We quantified population‐level heterogeneity (hereafter referred to simply as ‘heterogeneity’) after (Laliberté & Legendre, 2010), which is applicable to a wide range of systems and can deal with multiple traits and missing values (Olusoji et al., 2023). We undertook heterogeneity calculations in the context of two‐dimensional σ,ι trait space, in which susceptibility (σ) and infectiousness (ι) form the two axes.

We first calculated the abundance‐weighted centroid (c, hereafter referred to as the ‘centroid’) of the population, given simply as the population mean of each trait ($\bar{\sigma }$ and $\bar{\iota }$, Figure 2a,c). We then calculated the heterogeneity score, h, by finding the mean abundance‐weighted, Euclidean distance to the centroid (e.g. $z_{1}$ in Figure 2b, $z_{22}$ in Figure 2d) of all sub‐populations. We calculated initial heterogeneity using the initial abundances of the sub‐populations and final (equilibrium) heterogeneity using equilibrium sub‐population abundances.

**FIGURE 2:**
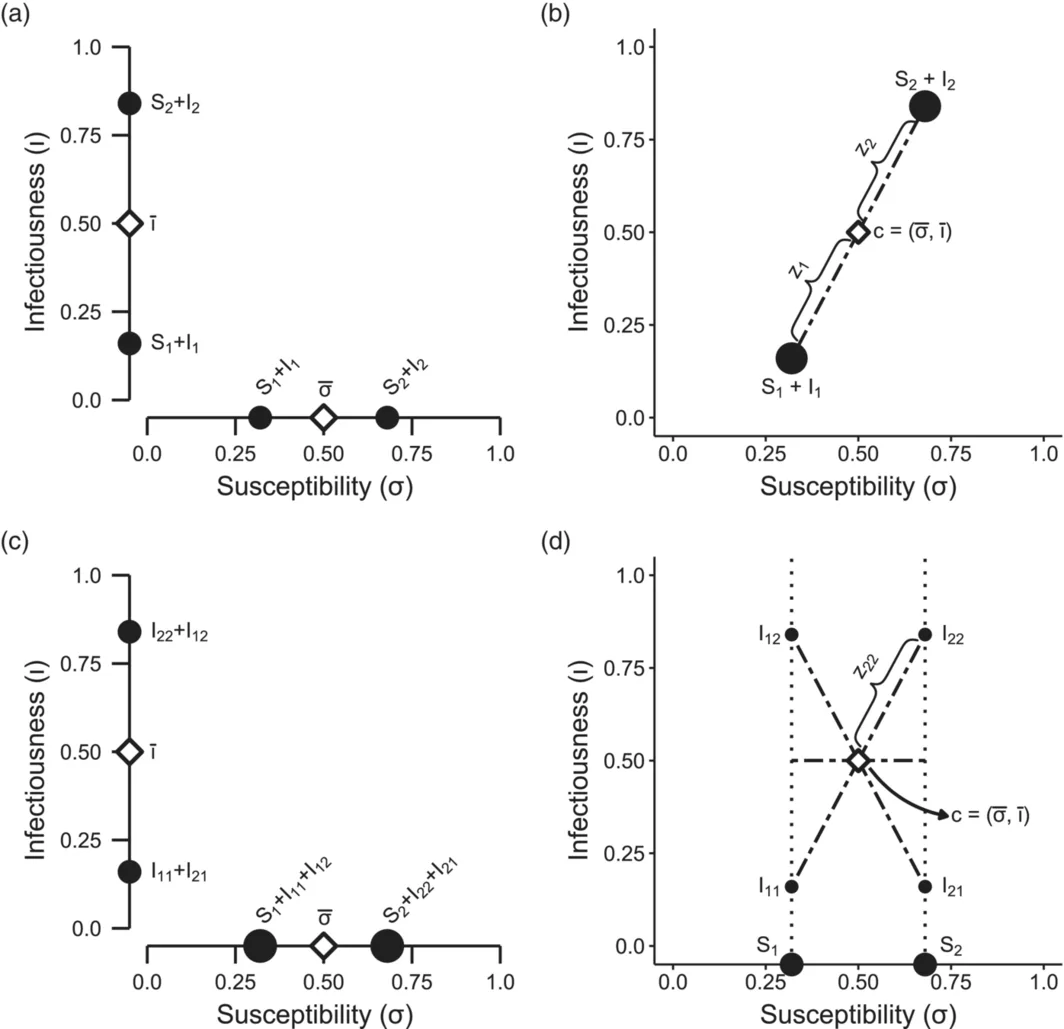
Schematic representation for calculating heterogeneity for the recipient dependence (RD) (a, b) and donor dependence (DD) (c, d) scenarios. For both the RD and DD scenarios, the population mean susceptibility ($\bar{\sigma }$) and population mean infectiousness ($\bar{\iota }$) are calculated in a single dimension (a, c). The sizes of the black dots indicate the relative abundances of the relevant sub‐populations. (b) Heterogeneity for the RD scenario is the mean Euclidean distance ($z_{j}$), weighted by the abundance of each sub‐population, to the centroid (c). (d) Heterogeneity for the DD scenario is the mean of the Euclidean distances ($z_{\mathrm{jk}}$) of the infected sub‐populations to the centroid, and the single dimension distance (σ) of the susceptible sub‐populations, weighted by the abundance of each sub‐population; here the values for $S_{1}$ and $S_{2}$ form vertical lines rather than points in σ,ι trait space because they have no infectiousness values. In all panels, the diameters of the black circles represent the abundance of the sub‐population.

The calculation of heterogeneity differs between the RD and DD scenarios in how sub‐populations were grouped, and abundances calculated. Specifically, for RD, all individuals in the same susceptible sub‐population move to the same infected sub‐population, and so we summed the abundances of the corresponding susceptible and infected sub‐populations. These grouped sub‐populations were then used to calculate h. For DD, each infected sub‐population's Euclidean distance to the centroid was calculated using both its σ and ι values (e.g. $I_{11}$ in Figure 2d) while, because susceptible sub‐populations only had a σ value, their distance to the centroid was calculated in a single dimension (represented by a vertical dotted line, e.g. $S_{1}$ in Figure 2d), and so there were no grouped sub‐populations used in calculating h. The formulae for heterogeneity calculations for both the RD and DD scenarios can be found in the Supporting Information.

### 2.5 Model analyses

We analysed both the RD and DD scenarios under three heterogeneity contexts:

1. *Bipartite heterogeneity,* where the population is divided into two sub‐populations corresponding to two distinct pairs of susceptibility and infectiousness values. This minimal structure allows variation in population‐level heterogeneity and covariance in a controlled manner, while maintaining analytical transparency
2. *Tripartite isometric heterogeneity,* where the population is divided into three sub‐populations corresponding to three distinct pairs of susceptibility and infectiousness values, equidistant from each other in σ,ι trait space. This structure allows heterogeneity to increase while maintaining zero covariance between traits. This provides a stringent test of whether results observed under bipartite heterogeneity depend on the covariance structure.
3. *Tripartite non‐isometric heterogeneity,* where the population is divided into three sub‐populations corresponding to three distinct pairs of susceptibility and infectiousness values, but which are not necessarily equidistant in σ,ι trait space. This more general structure relaxes the symmetry assumptions and allows heterogeneity and covariance to vary independently, enabling a test of the robustness of the results from bipartite heterogeneity.

Here we present analyses relating to context 1; contexts 2 and 3 are presented in the Supporting Information.

For the bipartite heterogeneity context, we set the initial number of susceptibles per sub‐population to 49 and the number of infecteds per sub‐population to 1 (RD, 2 sub‐populations) or 0.5 (DD, 4 sub‐populations). Initial mean population susceptibility and infectiousness trait values were 0.5; thus $\sigma_{2}=1-\sigma_{1}$ and $\iota_{2}=1-\iota_{1}$. All other parameter values were the same for all analyses (Table 1). By varying $\sigma_{1}$ and $\iota_{1}$, we varied the initial population heterogeneity while maintaining the same initial mean population trait values. Hence, the effects of changing heterogeneity were decoupled from the effects of changing initial mean population trait values.

**TABLE 1:** Parameter definitions and values for all analyses of the RD and DD models.

Model parameters	Definition	Value
κ	Contact rate per individual per time	0.5
b	Birth rate per time	1.5
d	Mortality rate per time	1
α	Parasite‐induced mortality rate per time	0.7

We generated 2601 unique combinations of σ and ι trait values, and used these to analyse both the RD and DD models to understand how heterogeneity in susceptibility and infectiousness affected three key descriptors of epidemiological dynamics: (i) the basic reproduction number (*R*_0_), a measure of epidemic potential (Anderson & May, 1991); (ii) the equilibrium host population abundance, which quantifies the impact of the parasite on the host population; and (iii) the change in heterogeneity between the initial and final state of the system, indicating how heterogeneity changes with epidemic progression (presented in the Supporting Information).

### 2.6 Calculating *R*_0_

*R*_0_ predicts the risk of an epidemic occurring, as well as the size and severity of that epidemic (Anderson & May, 1991; Heffernan et al., 2005), and also the effort needed to control and eliminate a parasite from a population (Roberts, 2007). Thus, understanding the effect of heterogeneity on *R*_0_ provides considerable insight into how host heterogeneity in susceptibility and infectiousness affects parasite transmission through a population.

Using next generation matrices (Diekmann et al., 2010) (shown in full in the Supporting Information), and assuming that infecteds are initially rare, we calculated *R*_0_ for each scenario to be

**RD:**

(11) $$\begin{array}{c} R_{0}=\frac{\mathrm{\kappa N}}{d+\alpha }\left(\mathrm{cov}\left(\sigma ,\iota \right)+\bar{\sigma }\;\bar{\iota }\right), \end{array}$$

**DD:**

(12) $$\begin{array}{c} R_{0}=\frac{\kappa \iota_{\max}\bar{\sigma }N}{d+\alpha }, \end{array}$$

where $\iota_{\max}$ is the maximum infectiousness value across all sub‐populations.

### 2.7 Equilibrium analyses

When possible we calculated the equilibrium solutions of Equations ((5), (6), (7), (8)) analytically. For parameter values for which this was not possible, we solved the system numerically over 50,000 time steps, which was sufficiently long to reach equilibrium. Any sub‐population with susceptibility σ=0 (i.e. completely resistant to infection) experiences unbounded growth, and so such cases were omitted from equilibrium analyses.

We also used these equilibrium solutions to calculate equilibrium population‐level heterogeneity (following the method described above) to test whether the population‐level heterogeneity changed with epidemiological progress.

### 2.8 Software packages

Plots were generated in R (R Core Team, 2022) using packages ggplot2 (Wickham, 2016), ggforce (Pedersen, 2022a), scales (Wickham & Seidel, 2022), showtext (Qiu, 2022), pBrackets (Schulz, 2021), patchwork (Pedersen, 2022b) and latex2exp (Meschiari, 2022). Equilibrium analyses were conducted using Mathematica (Wolfram Research Inc., 2022). Heterogeneity and *R*_0_ values were calculated in R (R Core Team, 2022).

## 3 RESULTS

Here we present analysis of the bipartite heterogeneity context, where there are two sub‐populations. The results for the tripartite heterogeneities are broadly consistent with the bipartite analyses, highlighting the generality of the findings; we describe those results in the Supporting Information.

### 3.1 How heterogeneity affects *R*_0_ under RD versus DD scenarios

#### 3.1.1 Recipient dependence

Equation (11) shows explicitly that, under RD, heterogeneity affects *R*_0_ through the covariance between susceptibility and infectiousness; positive covariance between susceptibility and infectiousness increases *R*_0_ relative to the homogeneous population, negative covariance reduces *R*_0_ and variation in only one trait leaves *R*_0_ unchanged. These relationships are shown in Figure 3a,b, where positive covariance (red points) have higher *R*_0_ values and negative covariance (blue points) have lower *R*_0_ values. At high levels of heterogeneity, the distribution of *R*_0_ values bifurcates due to the boundaries of σ,ι trait space that restrict the number of viable trait combinations (Figure 3a). For example, with maximum heterogeneity there are only two possible configurations of the sub‐populations such that they lie at the opposite extremes of the diagonals in σ,ι trait space, resulting in just two points (Figure 3a).

**FIGURE 3:**
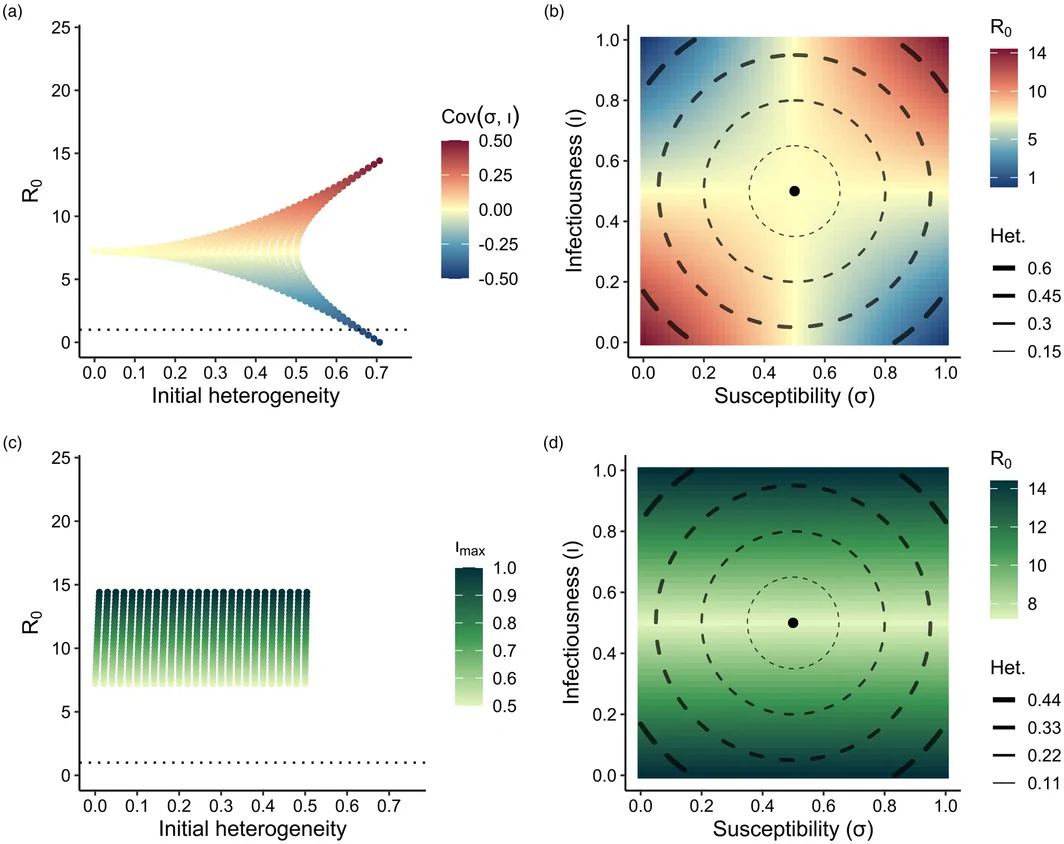
The effect of initial heterogeneity on *R*_0_ for the recipient dependence (RD) (a, b) and donor dependence (DD) (c, d) scenarios. The dotted line in (a) and (c) is where *R*_0_ = 1. For RD (a), *R*_0_ changes as initial heterogeneity increases, and with the covariance between susceptibility (σ) and infectiousness (ι), as indicated by the colour bar. (b) shows *R*_0_ (the colour scale) plotted in σ,ι trait space with the centroid (c) at σ=ι=0.5, and concentric dashed‐line circles showing increasing heterogeneity; the positions of the sub‐populations are mirrored across the centroid ($\sigma_{2}=1-\sigma_{1}$ and $\iota_{2}=1-\iota_{1}$). For DD (c), *R*_0_ does not change as initial heterogeneity increases, but scales with maximum infectiousness ($\iota_{\max}$), as the green colour bar. (d) shows the DD version of panel (b), but note that the *R*_0_ and heterogeneity scales differ between (b) and (d).

In summary, increasing heterogeneity in the RD scenario can lead to increasingly divergent *R*_0_, compared to the homogeneous population. But, whether *R*_0_ is greater or less than the homogeneous population is determined by whether the population‐level covariance between susceptibility and infectiousness is positive or negative, respectively. When the covariance is zero (heterogeneity in either susceptibility or infectiousness, but not both), *R*_0_ is the same as the homogeneous population, regardless of the degree of heterogeneity.

#### 3.1.2 Donor dependence

In the DD scenario, *R*_0_ is affected in a qualitatively different manner; specifically, *R*_0_ is determined by the maximum infectiousness present in the population (Figure 3c, Equation 12), and can therefore be written directly in terms of an extreme value rather than an expectation across host classes. As a result, increasing heterogeneity does not reduce *R*_0_ relative to the homogeneous population, and variation in susceptibility alone has no effect on *R*_0_ when susceptible sub‐populations are of equal size (Figure 3c,d). For the same overall degree of heterogeneity (i.e. vertical slice in Figure 3c), increasing heterogeneity in infectiousness (and a necessary decrease in heterogeneity in susceptibility) increases *R*_0_. Susceptibility can only affect *R*_0_ when the initial susceptible sub‐population abundances are not equal (see Supporting Information).

#### 3.1.3 Summary of effects of heterogeneity on R_0_

In both the RD and DD, scenarios increasing heterogeneity can lead to increasingly divergent *R*_0_ values compared to the homogeneous case. However, what drives this effect on *R*_0_ differs between the two scenarios. In the RD scenario, it is the population‐level covariance between susceptibility and infectiousness; in the DD scenario it is the maximum infectiousness in the population. Sensitivity analyses of these results to changes in the key demographic parameters (b, d, κ and α) are presented in Figures S5–S8, showing qualitatively similar findings across parameter values.

### 3.2 How heterogeneity affects host abundance under RD versus DD scenarios

Equilibrium total host abundance generally increases with increasing initial heterogeneity, as does the variability in equilibrium abundance for both the RD and DD scenarios (Figure 4). However, specific aspects of these relationships differ between the two scenarios.

**FIGURE 4:**
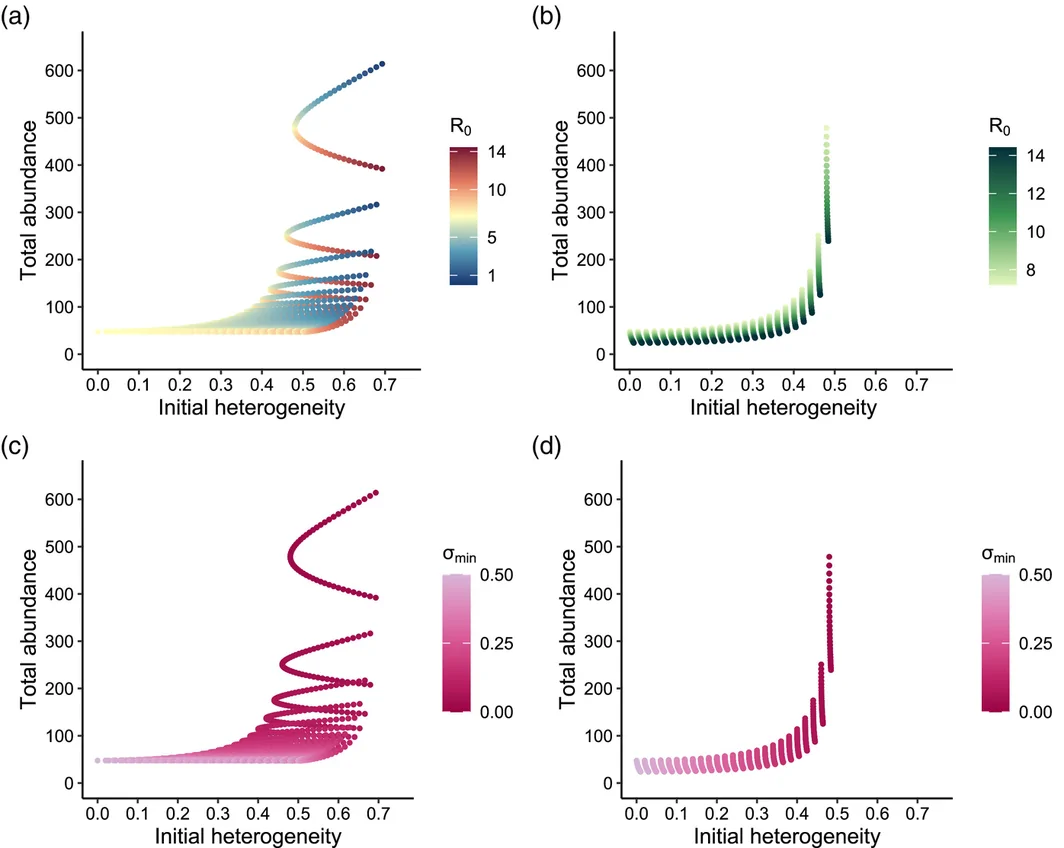
The effect of initial heterogeneity on equilibrium total host abundance for recipient dependence (RD) (a, c) and donor dependence (DD) (b, d) scenarios. (a) and (c) are the same RD plots, but differently coloured: (a) for *R*_0_ and therefore also the covariance between susceptibility and infectiousness, where red points indicate positive covariance and blue points indicate negative covariance; (c) for minimum population‐level susceptibility ($\sigma_{\min}$). (b) and (d) are the same DD plots, but differently coloured: (b) for *R*_0_; (d) for $\sigma_{\min}$. Note that the *R*_0_ scales differ between (a) and (b). An enlarged version of (a) and (c) can be found in the Supporting Information (Figure S2). Sensitivity analyses of these results to changes in the key demographic parameters (b, d, κ and α) are presented in Figures S7 and S8, again showing qualitatively similar findings across parameter values.

#### 3.2.1 Recipient dependence

In the RD scenario, there is a complex relationship between initial heterogeneity and host equilibrium abundance (Figure 4a,c, and see Figure S2 for an enlarged version at the lower host abundance range). Equilibrium abundances are grouped into parabolic ‘clusters’ where each cluster has equilibrium abundances that both increase and decrease from an initial baseline abundance as heterogeneity increases. Each cluster is determined by the minimum susceptibility in the population; all points in a cluster have the same population‐level minimum susceptibility value (and so also the same maximum susceptibility value) (Figure 4c). Clusters are ordered by these minimum susceptibility values; those with the lowest minimum susceptibility values ($\sigma_{\min}$, Figure 4c) have the highest abundances. This is because in populations with low minimum susceptibility fewer individuals become infected, so that fewer hosts are exposed to parasite‐induced mortality (α), which thereby increases overall host abundance.

The within‐cluster divergence in host abundance that occurs with increasing heterogeneity arises due to increasingly divergent infectiousness values in the population. Since all points within a cluster have the same pair of σ values for the component sub‐populations, an increase in heterogeneity is achieved by divergence in the two infectiousness values. This divergence, and so increase in heterogeneity, leads to changes in *R*_0_ within a cluster that changes equilibrium host abundance. High *R*_0_ values lead to lower abundances (the lower red tail of a cluster in Figure 4a), while low *R*_0_ values lead to higher abundances (the upper blue tail of a cluster in Figure 4a). Figure 4a,c include all parameter combinations of susceptibility (σ) and infectiousness (ι), and so both positive and negative covariance between the two traits. Thus, within a given cluster, positive covariance (high *R*_0_; red colours) produces lower equilibrium host abundance, whereas negative covariance (low *R*_0_; blue colours) produces higher equilibrium host abundance.

#### 3.2.2 Donor dependence

In the DD scenario, there are also clusters in equilibrium host abundance determined by the minimum susceptibility value ($\sigma_{\min}$) within the population (Figure 4d), but the points within each cluster are arranged in near‐vertical lines, suggesting that heterogeneity has little effect on equilibrium host abundance within a cluster. As with the RD scenario, equilibrium host abundance is maximised when *R*_0_ is minimised (Figure 4b) which, for the DD, scenario occurs when the maximum infectiousness in the population ($\iota_{\max}$) is at its lowest (Figure 3c). Hence, within each cluster, increasing maximum infectiousness increases *R*_0_, reducing equilibrium host abundance. The DD scenario generally shows lower equilibrium host abundances than the RD scenario because the minimum *R*_0_ values for the RD scenario are lower than the DD scenario.

## 4 DISCUSSION

While existing theory shows that host heterogeneity in susceptibility and infectiousness can affect population‐level parasite transmission (e.g. Lloyd et al., 2020), our findings demonstrate that these effects differ considerably depending how host infectiousness is determined. A key contribution of this work is demonstrating that epidemiological outcomes depend not only on the presence of host heterogeneity, but on the way in which host infectiousness is determined. Previous work has often implicitly assumed that infectiousness is an intrinsic property of infected hosts (i.e. matching the RD scenario). By contrasting the RD and DD scenarios, we demonstrate that superficially similar forms of heterogeneity can lead to qualitatively different drivers of transmission. Specifically, we show that the contrasting RD and DD scenarios result in different relationships between host heterogeneity in susceptibility and infectiousness and epidemiological outcomes for both *R*_0_ and host abundance.

While we find that *R*_0_ changes with increasing heterogeneity in both scenarios, there is a notable contrast between the two scenarios in what drives these effects, and the direction of the effects. The RD scenario shows divergent *R*_0_ values as heterogeneity increases, which is determined by the covariance between susceptibility and infectiousness. This finding is consistent with previous modelling: three models of vector‐borne infections, where infectiousness was an inherent host trait (i.e. implicitly assuming RD), incorporated host heterogeneity in susceptibility and infectiousness and found that positive covariance between heterogeneities led to an increase in *R*_0_ relative to the homogeneous case, while negative covariance led to a decrease (Dietz, 1980; Koella, 1991; Vazquez‐Prokopec et al., 2016). Thus, these models showed that in a RD scenario, population‐level covariance between susceptibility and infectiousness determines *R*_0_, consistent with our findings.

In contrast, we find that in the DD scenario *R*_0_ is determined by the maximum infectiousness in the population, such that *R*_0_ increases as heterogeneity in infectiousness increases. Heterogeneity in susceptibility is largely irrelevant for the DD scenario, only becoming so if abundances are very different between sub‐populations. There are fewer other studies that consider DD‐like scenarios, but one example is the hypothesis that patterns of SARS‐CoV‐2 transmission may be due to superspreaders tending to generate new superspreaders, for example, through a dose‐dependent effect (Beldomenico, 2020). A model exploring how *R*_0_ responded to this scenario (compared to the null model where superspreaders appeared randomly) showed that *R*_0_ increases with an increase in the probability that a superspreader generates additional superspreaders (Wanelik et al., 2023). Thus, moving from the null model to a DD‐like scenario increased *R*_0_, suggesting that the DD scenario tends to increase *R*_0_, consistent with our findings.

The RD and DD scenarios also differ in how host heterogeneity affects equilibrium total host abundance. While host abundance tends to increase with increasing heterogeneity in both scenarios, the effect is more pronounced in the RD scenario than in the DD scenario. Further, for a given susceptibility value, RD and DD differ by generating divergent (RD) or monotonic (DD) changes in equilibrium host abundance, a pattern that becomes more pronounced with increasing heterogeneity. It should be noted that although infection prevalence is a commonly reported epidemiological outcome, we found that heterogeneity had no effect on equilibrium infection prevalence in either scenario (further details in Supporting Information).

These RD and DD scenarios are phenomenological characterisations that each encompass a variety of real‐world processes. Although few host–parasite systems are likely to represent pure RD, some systems exhibit a strong tendency towards RD, including canaries, where an individual's nutritional status can affect their infectiousness with avian malaria (Cornet et al., 2014); rabbit myxoma virus infection status determines its infectiousness for co‐infecting nematodes (Cattadori et al., 2007); several examples of different host strains exhibiting varying levels of infectiousness when infected with the same parasite isolates (Bolas‐Fernandez & Wakelin, 1989; Dorfman et al., 2024; Jørgensen et al., 1998); a genetic basis for observed variation in host infectiousness in *Scophthalmus maximus* (Turbot) infected with a ciliate parasite (Anacleto et al., 2019) as well as *Drosophila melanogaster* infected with Drosophila C Virus (Siva‐Jothy & Vale, 2021). In addition, critical to understanding parasite transmission in a RD scenario is covariance between susceptibility and infectiousness, and there are empirical examples of this. For example, it has been shown that juvenile *Cygnus columbianus bewickii* (Bewick's swan) are more susceptible to avian influenza virus than adults and also have higher shedding rates than adults, demonstrating positive covariance between host susceptibility and infectiousness (Hoye et al., 2012). Alternatively, negative covariance between host susceptibility and infectiousness has been proposed as a mechanism for social insects avoiding parasitism by investing more in anti‐parasitic behaviours to reduce their infectiousness in a colony with high susceptibility (Cremer et al., 2007), such that a highly susceptible colony would be less infectious than a less susceptible colony. At the individual level, negative covariance might occur when highly susceptible individuals acquire high levels of disease, thus increasing sickness behaviours that reduce host infectiousness (Araujo et al., 2016).

Likewise, some systems exhibit a strong tendency towards DD, such as calves infected with three different doses of bovine viral diarrhoea virus (BVDV), where the most infectious individuals were those given the highest viral dose, due to a longer infectious period (Strong et al., 2015). Similar patterns have been found with a number of other host–parasite systems (Gaskell & Povey, 1979; Mumford et al., 1990; Zarkov, 2012). Experimental comparisons of influenza virus strains in ferrets show that highly transmissible strains replicate faster and generate higher airborne viral RNA shedding than poorly transmissible strains, illustrating how the pathogen strain received at infection can influence subsequent transmission potential (Pulit‐Penaloza et al., 2023). In addition, analogous donor‐mediated processes have been explored in studies of *symptom propagation*, where traits expressed following infection depend on characteristics of the donor host (Asplin, Keeling, et al., 2024; Asplin, Mancy, et al., 2024).

In reality, we do not expect most host–parasite systems to correspond exactly to either pure RD or pure DD. Instead, many systems are likely to lie somewhere along a RD–DD continuum, with infectiousness influenced by both characteristics of the recipient host and characteristics of the donor host responsible for transmission. For example, in the BVDV calf infection study, calves infected with higher viral doses subsequently became more infectious (Strong et al., 2015), suggesting a strong DD component. However, infectiousness also varied among calves receiving the same dose, indicating that RD may also contribute. Conversely, although the myxoma‐nematode system in rabbits appears largely consistent with RD because infectiousness depends on characteristics of the infected host (Cattadori et al., 2007), DD effects may also occur if hosts receiving larger parasite doses subsequently become more infectious themselves. In addition, host genetic background and sex explained approximately 12% of variation in transmission potential in *D. melanogaster* infected with Drosophila C virus, suggesting that other factors, potentially including donor‐associated processes, may also contribute (Siva‐Jothy & Vale, 2021).

The examples in the preceding paragraph illustrate that most empirical systems are unlikely to be wholly RD or DD, but may instead be dominated by one mechanism while still retaining elements of the other. Hence, RD and DD should be viewed as limiting cases that isolate the epidemiological consequences of these alternative mechanisms. Understanding these limiting cases provides a foundation for interpreting more complex systems in which both processes operate simultaneously. In particular, our results suggest that identifying which mechanism predominates in a given system may be particularly important because the two limiting cases generate fundamentally different drivers of transmission. Under RD, *R*_0_ is governed by the covariance between susceptibility and infectiousness, whereas under DD, it is governed by the maximum infectiousness present in the population. Consequently, determining whether a system is primarily RD‐ or DD‐driven may provide insight into which population‐level characteristics are most important for predicting transmission and designing interventions.

The work we present here deliberately prioritises conceptual clarity over exhaustive parameter exploration. Nevertheless, to assess the robustness of our results we conducted a sensitivity analysis varying key demographic and epidemiological parameters. This showed that the qualitative contrasts between RD and DD are robust to reasonable parameter variation (details in Supporting Information). The contrast between the two scenarios fundamentally arises from how infectiousness is determined following infection and we would therefore also expect our findings to be robust to other modelling extensions. In particular, because RD and DD differ in how infectiousness is determined, rather than in the functional form of transmission itself, we expect their qualitative distinction to persist across alternative transmission formulations, including frequency‐dependent or nonlinear transmission. Exploring alternative transmission functions such as those described by McCallum et al. (2001), or higher dimensional heterogeneity are natural directions for future work. In addition, extending the framework to allow infectiousness to be jointly determined by recipient and donor characteristics would require explicit assumptions regarding how those contributions combine and how newly infected hosts are assigned to infectiousness classes. Because multiple biologically plausible formulations are possible, we focus here on the two limiting cases that isolate the consequences of each mechanism separately; developing mixed RD–DD models lies beyond the scope of the present study and represents an important direction for future work.

Our model also makes simplifying assumptions regarding host demography and infection dynamics, notably the absence of density‐dependent regulation and recovery from infection. Such processes are known to influence equilibrium outcomes in multi‐host and structured disease models (e.g. Dobson, 2004; Holt & Pickering, 1985). However, our aim was not to predict absolute population trajectories, but to compare how contrasting scenarios for determining infectiousness shape transmission dynamics. Because these assumptions apply equally to both scenarios, it is likely that they do not undermine the comparative conclusions of the study. Instead, incorporating density dependence or recovery might introduce additional feedbacks that may modify quantitative outcomes, though we believe that the qualitative distinction between RD and DD would likely be preserved. Further exploration of this topic could be an interesting avenue for future work.

Previous work has typically treated how a host's infectiousness is determined as a black box, generally assuming it is a fixed property of the recipient host. This overlooks the importance of how a host's infectiousness is determined in influencing the consequences of host heterogeneity for parasite transmission. Yet this process can have real‐world consequences. For instance, there is interest in breeding parasite resistant livestock to reduce the substantial economic and climatic costs caused by parasites in livestock systems (Knap & Doeschl‐Wilson, 2020). However, to determine the livestock traits that are most appropriate to select, it will be important to consider where the host–parasite system falls on the RD–DD spectrum. For example, in an RD scenario, selecting for reduced parasite susceptibility (i.e. for resistance) versus in a DD scenario selecting for reduced parasite shedding. The work we present here shows the importance of explicitly considering how infectiousness is determined and that ignoring it can lead to an incomplete understanding of the effects of host heterogeneities on parasite transmission. A failure to do so could have consequences for both future theoretical and empirical work.

## AUTHOR CONTRIBUTIONS

Jacob A. Cohen, Andy Fenton and Mark Viney jointly conceived the research. Jacob A. Cohen conducted the research and formal analysis. Andrew D. Dean contributed to the formal analysis. Jacob A. Cohen wrote the first draft of the manuscript, and all authors contributed to revisions.

## CONFLICT OF INTEREST STATEMENT

The authors declare no conflicts of interest for this work.

## Supporting information


**Figure S1.** Contours of constant heterogeneity in σ,ι trait space, with two pairs of trait values of equal population abundance.
**Figure S2.** Effect of initial heterogeneity on equilibrium total host abundance for RD, enlarged to focus on total abundances below 100.
**Figure S3.** Change in heterogeneity from the initial sub‐population values to their equilibrium values (the dotted line shows y=x in both panels).
**Figure S4.** Prevalence of infected hosts at equilibrium with initial heterogeneity.
**Figure S5.** Sensitivity analysis of the effect of host heterogeneity on *R*_0_ under RD (equivalent to Figure 3a in the main paper).
**Figure S6.** Sensitivity analysis of the effect of host heterogeneity on *R*_0_ under DD (equivalent to Figure 3c in the main paper).
**Figure S7.** Sensitivity analysis of the effect of host heterogeneity on equilibrium total host abundance under RD (equivalent to Figure 4a in the main paper).
**Figure S8.** Sensitivity analysis of the effect of host heterogeneity on equilibrium total host abundance under DD (equivalent to Figure 4b in the main paper).
**Figure S9.** The effect of initial heterogeneity on *R*_0_.
**Figure S10.** Effect of initial heterogeneity on total abundance of hosts at equilibrium.
**Figure S11.** Change in heterogeneity from the initial sub‐population values to their equilibrium values.

## Data Availability

Data are available from Zenodo https://doi.org/10.5281/zenodo.21340138 (Cohen et al., 2026).
