# Supplementary material for: The consequences of host heterogeneity for parasite transmission depend on how host infectiousness is determined
Source: J Anim Ecol. 2026 Jul 31;95(10):2063–75. doi: 10.1111/1365-2656.70329 (PMC13643203; doi:10.1111/1365-2656.70329)
Supplement: Supplementary file 1 [file JANE-95-2063-s001.docx]

**Supplementary Information**

**1. Calculation of heterogeneity scores**

We adapted the method outlined in (Laliberté & Legendre 2010) to calculate heterogeneity as the weighted mean of the Euclidean distances in trait space of each sub-population from the population mean, where the weights are given by the abundances of each sub-population. The following calculations are presented graphically in Figure 1 for the bipartite heterogeneity context $n=2$, $\sigma_{2}=1-\sigma_{1}$ and $\iota_{2}=1-\iota_{1}$.

***Recipient dependence***

In the RD scenario, there are $n$ susceptible and $n$ infected sub-populations, with the $j$th sub-populations sharing the same values of $\sigma$ and $\iota$. The population mean trait values are therefore

$$\begin{aligned} \bar{\sigma}=\frac{1}{N}\sum_{j=1}^{n} \sigma_{j}(S_{j}+I_{j}),\#(S1) \end{aligned}$$

$$\begin{aligned} \bar{\iota}=\frac{1}{N}\sum_{j=1}^{n} \iota_{j}\left( S_{j}+I_{j} \right).\#(S2) \end{aligned}$$

The Euclidean distance of the $j$th sub-population to the population mean is

$$\begin{aligned} z_{j}=\sqrt{\left( \bar{\sigma}-\sigma_{j} \right)^{2}+\left( \bar{\iota}-\iota_{j} \right)^{2}},\#(S3) \end{aligned}$$

and so population heterogeneity is given by

$$\begin{aligned} h=\frac{1}{N}\sum_{j=1}^{n} z_{j}(S_{j}+I_{j}).\#(S4) \end{aligned}$$

***Donor dependence***

In the DD scenario, there are $n_{S}$ susceptible sub-populations, each with no infectiousness trait, and $n_{S}n_{I}$ infected sub-populations, each with both traits. The population mean trait values are therefore

$$\begin{aligned} \bar{\sigma}=\frac{1}{N}\sum_{j=1}^{n_{S}} \sigma_{j}\left( S_{j}+\sum_{k=1}^{n_{I}} I_{j,k} \right),\#(S5) \end{aligned}$$

$$\begin{aligned} \bar{\iota}=\frac{1}{\sum_{k=1}^{n_{I}} \sum_{j=1}^{n_{S}} I_{j,k}}\sum_{k=1}^{n_{I}} \sum_{j=1}^{n_{S}} \iota_{k}I_{j,k}.\#(S6) \end{aligned}$$

Note that the population size for calculating the mean infectiousness is the sum of all infected sub-populations, not $N$. The Euclidean distance for the susceptible sub-populations in this case is simply $z_{j}=\left| \bar{\sigma}-\sigma_{j} \right|$ (see Figure 1D), while for the infectious sub-populations it is

$$\begin{aligned} z_{j,k}=\sqrt{\left( \bar{\sigma}-\sigma_{j} \right)^{2}+\left( \bar{\bar{\iota}}-\iota_{k} \right)^{2}}.\#\left( S7 \right) \end{aligned}$$

Hence population heterogeneity is given by

$$\begin{aligned} h=\frac{1}{N}\left( \sum_{j=1}^{n_{S}} \left( z_{j}S_{j}+\sum_{k}^{n_{I}} z_{j,k}I_{j,k} \right) \right).\#\left( S8 \right) \end{aligned}$$

**2. *R_0_* calculation through next generation matrices**

To calculate *R_0_*, we followed the method described in (Diekmann *et al.* 2010), in which *R_0_* is given by the dominant eigenvalue of the next-generation matrix $K=-T\Sigma^{-1}$, where $T$ comprises the rates of the infection transmission events in the infected subsystem, and $\Sigma$ the rates of the transition (e.g. mortality) events.

***Recipient dependence***

In the RD scenario, the infected subsystem is Equation (6). In this subsystem, the element in the $x$th row and $y$th column of the transmission matrix is

$$\begin{aligned} T_{xy}=\kappa\sigma_{x}S_{x}\iota_{y}, 1\leq x\leq n, 1\leq y\leq n,\#\left( S9 \right) \end{aligned}$$

and the transition matrix is $\Sigma=-\left( d+\alpha\right)I_{n}$, where $I_{n}$ is the $n$-dimensional identity matrix. Thus, the elements of the next-generation matrix are

$$\begin{aligned} K_{xy}=\frac{\kappa\sigma_{x}S_{x}\iota_{y}}{d+\alpha}, 1\leq x\leq n, 1\leq y\leq n.\#\left( S10 \right) \end{aligned}$$

Now, $K$ has rank 1, because each row is a scalar multiple of $\left( \iota_{1},\ldots,\iota_{n} \right)$, and therefore has precisely one non-zero eigenvalue. Because the sum of the eigenvalues of a matrix is equal to its trace, we therefore have

$$\begin{aligned} R_{0}=\frac{\kappa}{d+\alpha}\sum_{j=1}^{n} \sigma_{j}\iota_{j}S_{j}.\#\left( S11 \right) \end{aligned}$$

The covariance of the two trait values is

$$\begin{aligned} \mathrm{cov}\left( \sigma,\iota\right)=\frac{1}{N}\sum_{j}^{n} \sigma_{j}\iota_{j}\left( S_{j}+I_{j} \right)-\bar{\sigma} \bar{\iota};\#\left( S12 \right) \end{aligned}$$

hence, if $N\gg I_{j}$, as at the beginning of an infection, we can write

$$\begin{aligned} R_{0}\approx\frac{\kappa N}{d+\alpha}\left( \mathrm{cov}\left( \sigma,\iota\right)+\bar{\sigma} \bar{\iota} \right).\#\left( S13 \right) \end{aligned}$$

In other words, *R_0_* scales with the covariance of $\sigma$ and $\iota$, as seen in Figure 2 for $n=2$.

***Donor dependence***

In the DD scenario, the infected subsystem is Equation (10); note we use the system as written in terms of $I_{k}$, not $I_{j,k}$ as the susceptibility of infected classes does not impact parasite transmission. In this subsystem, the transmission matrix is given by

$$\begin{aligned} T=\kappa\sum_{j=1}^{n_{S}} \sigma_{j}S_{j}\mathrm{diag}\left( \boldsymbol{\iota}_{DD} \right),\#\left( S14 \right) \end{aligned}$$

where $\boldsymbol{\iota}_{DD}=(\iota_{1},\ldots,\iota_{n_{I}})$ is the vector of infectiousness values and $\mathrm{diag}(\boldsymbol{x})$ is the diagonal matrix with $\boldsymbol{x}$ on the diagonal and zeroes elsewhere. The transition matrix is $\Sigma=-\left( d+\alpha\right)I_{n_{I}}$, and so the next-generation matrix is

$$\begin{aligned} K=\frac{\kappa}{d+\alpha}\sum_{j=1}^{n_{S}} \sigma_{j}S_{j}\mathrm{diag}\left( \boldsymbol{\iota}_{DD} \right).\#\left( S15 \right) \end{aligned}$$

The eigenvalues of a diagonal matrix are simply its diagonal entries, and so in this case the maximal eigenvalue is

$$\begin{aligned} R_{0}=\frac{\kappa\iota_{\max}}{d+\alpha}\sum_{j=1}^{n_{S}} \sigma_{j}S_{j},\#\left( S16 \right) \end{aligned}$$

where $\iota_{\max}$ is maximum infectiousness value present in the system. The population level mean of $\sigma$ is

$$\begin{aligned} \bar{\sigma}=\frac{1}{N}\left( \sum_{j=1}^{n_{S}} \sigma_{j}S_{j}+\sum_{k=1}^{n_{I}} \iota_{k}I_{k} \right);\#\left( S17 \right) \end{aligned}$$

hence, if $N\gg I_{k}$, as at the beginning of an infection, we can write,

$$\begin{aligned} R_{0}\approx\frac{\kappa\iota_{\max}\bar{\sigma}N}{d+\alpha}.\#\left( S18 \right) \end{aligned}$$

In main text Figure 3 (where $n=2$) we have equal abundances for susceptible sub-populations and $\sigma_{2}=1-\sigma_{1}$, so it is $\iota_{\max}$ alone that determines how *R_0_* varies in trait space.

**3. *R_0_* with bipartite heterogeneity**

In this section, we present a mathematical analysis of *R_0_* when there two different values of susceptibility and infectiousness, *i.e.* $n=2$ in RD and $n_{S}=n_{I}=2$ in DD.

***Recipient dependence***

In this case, we define $N_{j}=S_{j}+I_{j}$, $j=1,2.$ From Equation (S4), we can therefore write population heterogeneity as

$$\begin{aligned} h=\frac{N_{1}}{N}\sqrt{\left( \bar{\sigma}-\sigma_{1} \right)^{2}+\left( \bar{\iota}-\iota_{1} \right)^{2}}+\frac{N_{2}}{N}\sqrt{\left( \bar{\sigma}-\sigma_{2} \right)^{2}+\left( \bar{\iota}-\iota_{2} \right)^{2}}.\#\left( S19 \right) \end{aligned}$$

Using the definition of trait means (Equations (S1)-(S2)), this can be rewritten as

$$h=\frac{N_{1}}{N}\sqrt{\left( \bar{\sigma}-\sigma_{1} \right)^{2}+\left( \bar{\iota}-\iota_{1} \right)^{2}}+\frac{N_{2}}{N}\sqrt{\left( \bar{\sigma}-\frac{\bar{\sigma}N-\sigma_{1}N_{1}}{N_{2}} \right)^{2}+\left( \bar{\iota}-\frac{\bar{\iota}N-\iota_{1}N_{1}}{N_{2}} \right)^{2}}$$

$$=\frac{N_{1}}{N}\sqrt{\left( \bar{\sigma}-\sigma_{1} \right)^{2}+\left( \bar{\iota}-\iota_{1} \right)^{2}}+\frac{1}{N}\sqrt{\left( -\bar{\sigma}N_{1}+\sigma_{1}N_{1} \right)^{2}+\left( -\bar{\iota}N_{1}+\iota_{1}N_{1} \right)^{2}}$$

$$\begin{aligned} =\frac{2N_{1}}{N}\sqrt{\left( \bar{\sigma}-\sigma_{1} \right)^{2}+\left( \bar{\iota}-\iota_{1} \right)^{2}}.\#\left( S20 \right) \end{aligned}$$

Thus $N_{1}z_{1}=N_{2}z_{2}$, and Equation (S20) describes a circle in $\sigma_{1},\iota_{1}$ space, centred at trait means and with radius $hN/2N_{1}.$ We therefore define the polar coordinates

$$\begin{aligned} \sigma_{1}=\bar{\sigma}+\frac{\rho N}{2N_{1}}\cos\theta,\#(S21) \end{aligned}$$

$$\begin{aligned} \iota_{1}=\bar{\iota}+\frac{\rho N}{2N_{1}}\sin\theta,\#\left( S22 \right) \end{aligned}$$

and fix trait means at $\bar{\sigma}$ and $\bar{\iota}$. Hence, Equations (S1)-(S2) yield

$$\begin{aligned} \sigma_{2}=\bar{\sigma}-\frac{\rho N}{2N_{2}}\cos\theta,\#(S23) \end{aligned}$$

$$\begin{aligned} \iota_{2}=\bar{\iota}-\frac{\rho N}{2N_{2}}\sin\theta.\#\left( S24 \right) \end{aligned}$$

Note that $\rho$and $\theta$are implicitly restricted by the requirement that $0\leq\sigma_{j}\leq1$and $0\leq\iota_{j}\leq1.$ Under this coordinate transformation, we simply have $h=\rho$, *i.e.* contours of constant heterogeneity are circles centred on $\bar{\sigma}, \bar{\iota}.$

We now turn our attention to *R_0_*, assuming that $S_{j}\gg I_{j}$ and so $N_{j}\approx S_{j}.$In our polar coordinates, *R_0_* becomes

$$R_{0}\approx\frac{\kappa}{d+\alpha}\left( \sigma_{1}\iota_{1}N_{1}+\sigma_{2}\iota_{2}N_{2} \right)$$

$$=\frac{\kappa}{d+\alpha}\left( \left( \bar{\sigma}+\frac{\rho N}{2N_{1}}\cos\theta\right)\left( \bar{\iota}+\frac{\rho N}{2N_{1}}\sin\theta\right)N_{1}+\left( \bar{\sigma}-\frac{\rho N}{2N_{2}}\cos\theta\right)\left( \bar{\iota}-\frac{\rho N}{2N_{2}}\sin\theta\right)N_{2} \right)$$

$$=\frac{\kappa N}{d+\alpha}\left( \bar{\sigma}\bar{\iota}+\frac{\rho^{2}}{4}\cos\theta\sin\theta\left( \frac{N}{N_{1}}+\frac{N}{N_{2}} \right) \right)$$

$$\begin{aligned} =\frac{\kappa N}{d+\alpha}\left( \bar{\sigma}\bar{\iota}+\frac{\rho^{2}}{8}\sin2\theta\left( \frac{N}{N_{1}}+\frac{N}{N_{2}} \right) \right).\#\left( S25 \right) \end{aligned}$$

For fixed $\theta$, then, *R_0_* is monotonically increasing or decreasing with $\rho$. Differentiating with respect to $\theta$, we have

$$\begin{aligned} \frac{\partial R_{0}}{\partial\theta}=\frac{\kappa N}{d+\alpha}\frac{\rho}{4}\cos2\theta\left( \frac{N}{N_{1}}+\frac{N}{N_{2}} \right),\#\left( S26 \right) \end{aligned}$$

which vanishes when $\theta=\frac{\pi}{4},\frac{3\pi}{4},\frac{5\pi}{4},\frac{9\pi}{4}$. Differentiating *R_0_* with respect to $\theta$ again and evaluating at these zeros shows that the first and third of these are maxima and the second and fourth are minima. Thus *R_0_* is maximal for fixed heterogeneity on the positive diagonal $\theta=\frac{\pi}{4},\frac{5\pi}{4},$ centred on the trait means, in which case we have

$$\begin{aligned} \sigma_{1}-\bar{\sigma}=\iota_{1}-\bar{\iota}=\frac{\rho}{2\sqrt{2}N_{1}},\#\left( S27 \right) \end{aligned}$$

*i.e.* the two traits lie an equal distance in the same direction from the trait means (Figure S1). On the other hand, *R_0_* is minimal for fixed heterogeneity on the negative diagonal $\theta=\frac{3\pi}{4},\frac{9\pi}{4},$ in which case we have

$$\begin{aligned} \sigma_{1}-\bar{\sigma}=-\left( \iota_{1}-\bar{\iota} \right)=\frac{\rho}{2\sqrt{2}N_{1}},\#\left( S28 \right) \end{aligned}$$

*i.e.* the two traits lie an equal distance from, but in opposite directions from, the trait means (Figure S1).

Figure S1. Contours of constant heterogeneity in $\sigma,\iota$ trait space, with two pairs of trait values of equal population abundance. Contours are centred at trait means $\bar{\sigma}=0.5$, $\bar{\iota}=0.5$. The positive diagonal $\theta=\frac{\pi}{4},\frac{5\pi}{4}$ represents maximal values of *R_0_* on each contour, while the negative diagonal $\theta=\frac{3\pi}{4},\frac{9\pi}{4}$ represents minimal values of *R_0_*.

***Donor dependence***

In this case we assume that $N\approx S_{1}+S_{2}$, as we assume the infected sub-populations are small, so can re-write Equation S16 as

$$\begin{aligned} R_{0}=\frac{\kappa\iota_{\max}}{d+\alpha}\sum_{j=1}^{n_{S}} \sigma_{j}S_{j}.\#(S29) \end{aligned}$$

We then have

$$h\approx\frac{1}{N}\left| \bar{\sigma}-\sigma_{1} \right|S_{1}+\frac{1}{N}\left| \bar{\sigma}-\sigma_{2} \right|S_{2}$$

$$=\frac{1}{N}\left| \bar{\sigma}-\sigma_{1} \right|S_{1}+\frac{1}{N}\left| \bar{\sigma}-\frac{\bar{\sigma}N-\sigma_{1}S_{1}}{S_{2}} \right|S_{2}$$

$$\begin{aligned} =\frac{2}{N}\left| \bar{\sigma}-\sigma_{1} \right|S_{1}.\#\left( S30 \right) \end{aligned}$$

Hence contours of constant heterogeneity form (approximately) vertical lines in $\sigma_{1},\iota_{1}$ space.

From Equation (12), we have

$$\begin{aligned} R_{0}=\frac{\kappa\bar{\sigma}N}{d+\alpha}\max\left( \iota_{1},\frac{1}{I_{2}}(\bar{\iota}\left( I_{1}+I_{2} \right)-\iota_{1}I_{1} \right),\#\left( S31 \right) \end{aligned}$$

where we have used Equation (S6) to write $\iota_{2}$ in terms of $\bar{\iota}$. Now, $\max\left( \iota_{1},\frac{1}{I_{2}}(\bar{\iota}\left( I_{1}+I_{2} \right)-\iota_{1}I_{1} \right)=\iota_{1}$ only if $\iota_{1}\geq\bar{\iota}$, so in this case *R_0_* increases monotonically as $\iota_{1}$ moves away from $\bar{\iota}$ in either direction, as seen in Figure 2. The case shown in Figure 2 has $\bar{\sigma}$ fixed at 0.5, so there is no variation in the $\sigma_{1}$ direction.

**3. Bipartite heterogeneity results**

***RD host abundance***

Enlarging Figure 3 (main text) shows that total abundance is at the lowest values when the minimum population-level susceptibility value ($\sigma_{min}$) is at its highest, regardless of the amount of heterogeneity (Figure S2B).

Figure S2. Effect of initial heterogeneity on equilibrium total host abundance for RD, enlarged to focus on total abundances below 100. In (A) the colour bar shows *R_0_* values. In (B) the colour bar shows the minimum population-level susceptibility value ($\sigma_{min}$).

***Change in heterogeneity***

To understand how epidemic progress affects population-level heterogeneity we compared initial heterogeneity and its value at equilibrium. For both the RD and DD scenarios there is generally very little change in population-level heterogeneity throughout the epidemic (Figure S3).

Counterintuitively, we did not find a noticeable divergence between the RD and DD scenarios in how population-level heterogeneity changed during an epidemic. In the DD scenario we expected to see a loss of heterogeneity over time because the infected sub-population with the highest infectiousness value in the population becomes dominant as the epidemic progresses, ultimately excluding less infectious donors. However, heterogeneity is calculated by taking the mean abundance-weighted distance of the sub-populations to the centroid. Thus, despite the DD scenario losing infected sub-populations at equilibrium (and leading to maximally infectious hosts over time), the weighting of the heterogeneity score with the generally larger susceptible sub-populations ensures that there is no considerable loss in heterogeneity at equilibrium.

Figure S3. Change in heterogeneity from the initial sub-population values to their equilibrium values (the dotted line shows $y=x$ in both panels). (A) RD scenario, (B) DD scenario. Each point represents one simulation; points are light grey, such that darker points represent multiple, overlapping points.

***Infected host prevalence at equilibrium***

Figure S4. Prevalence of infected hosts at equilibrium with initial heterogeneity. A) RD model, B) DD model. In both cases, prevalence stays at ~71.43% regardless of the amount of heterogeneity.

***Sensitivity analyses***

To assess the robustness of our results to uncertainty in demographic and epidemiological parameters, we conducted a one-factor-at-a-time (OFAT) sensitivity analysis. Starting from the baseline parameter set used in the main analyses (main text Table 1), we varied each of the parameters governing host demography and transmission ($b$, $d$, $\kappa$, and $\alpha$) individually while holding all other parameters constant. For each parameter, two perturbations were considered, corresponding to multiplicative factors of 0.7 and 1.3 relative to the baseline value, yielding eight sensitivity runs per model (RD or DD), or 16 runs in total.

To ensure stable model dynamics, we enforced the constraint $d<b<d+\alpha$, ensuring that uninfected hosts had positive net demographic growth while infected hosts experienced net demographic decline. When a parameter perturbation violated this inequality, the varied parameter was adjusted minimally to satisfy the constraint, while all other parameters remained unchanged.

For each sensitivity run, equilibrium outcomes were computed using the same analytical and numerical procedures as in the baseline analysis. We compared results from the sensitivity analyses to baseline results for the effects of host heterogeneity on *R_0_* (RD Figure S5; DD Figure S6) and host abundance (RD Figure S7; DD Figure S8). Results from our sensitivity analyses confirm that the qualitative differences between RD and DD are robust to reasonable parameter variation.

It should be noted that the apparent differences between some panels of Figure S7 and Figure 4A (main text) are due to the altered parameters influencing how strongly changes in transmission translate into changes in host abundance, modifying the quantitative relationship between heterogeneity and equilibrium abundance. As a result, the visual structure of the heterogeneity-abundance relationship can differ between panels. For instance in some panels (Figure S7B, C & G), the range of equilibrium abundances is much larger than in the main analysis, such that within-cluster variation is visually compressed and appears approximately linear at the scale shown, even though the actual pattern is unchanged. The key organising principles – clustering by minimum susceptibility and the inverse relationship between abundance and *R_0_* – therefore remain intact across all sensitivity analyses. Thus, the same qualitative patterns are preserved across all sensitivity analyses. In every panel, simulations continue to group according to the minimum susceptibility present in the population, and equilibrium host abundance remains higher in simulations with lower *R_0_*.

Figure S5. Sensitivity analysis of the effect of host heterogeneity on *R_0_* under RD (equivalent to Fig 3A in the main paper). Each panel shows the relationship between initial heterogeneity and *R_0_* for one parameter perturbation, where demographic and transmission parameters ($b$, $d$, $\kappa$, or $\alpha$) were varied individually by multiplicative factors of 0.7 (left column) or 1.3 (right column) relative to their baseline values, while all other parameters were held constant. Rows correspond to the parameter varied, as indicated top right of each panel. Points are coloured by the covariance between susceptibility and infectiousness.

Figure S6. Sensitivity analysis of the effect of host heterogeneity on *R_0_* under DD (equivalent to Fig 3C in the main paper). Each panel shows the relationship between initial heterogeneity and *R_0_* for one parameter perturbation, where demographic and transmission parameters ($b$, $d$, $\kappa$, or $\alpha$) were varied individually by multiplicative factors of 0.7 (left column) or 1.3 (right column) relative to their baseline values, while all other parameters were held constant. Rows correspond to the parameter varied, as indicated top right of each panel. Points are coloured by the covariance between susceptibility and infectiousness.

Figure S7. Sensitivity analysis of the effect of host heterogeneity on equilibrium total host abundance under RD (equivalent to Fig 4A in the main paper). Each panel shows the relationship between initial heterogeneity and equilibrium total host abundance for one parameter perturbation, where demographic and transmission parameters ($b$, $d$, $\kappa$, or $\alpha$) were varied individually by multiplicative factors of 0.7 (left column) or 1.3 (right column) relative to their baseline values, while all other parameters were held constant. Rows correspond to the parameter varied, as indicated top right of each panel. Points are coloured by the covariance between susceptibility and infectiousness.

Figure S8. Sensitivity analysis of the effect of host heterogeneity on equilibrium total host abundance under DD (equivalent to Fig 4B in the main paper). Each panel shows the relationship between initial heterogeneity and equilibrium total host abundance for one parameter perturbation, where demographic and transmission parameters ($b$, $d$, $\kappa$, or $\alpha$) were varied individually by multiplicative factors of 0.7 (left column) or 1.3 (right column) relative to their baseline values, while all other parameters were held constant. Rows correspond to the parameter varied, as indicated top right of each panel. Points are coloured by the covariance between susceptibility and infectiousness.

**4. Tripartite heterogeneity results**

The results for the analyses of the tripartite heterogeneity contexts 2 and 3 are presented here. For each context we generated a range of unique combinations of $\sigma$ and $\iota$ trait values (n = 8,089 tripartite isometric heterogeneity, n = 109,561 tripartite non-isometric heterogeneity). The results are generally consistent with those from the bipartite heterogeneity analyses, particularly in the case of the tripartite non-isometric heterogeneity results, and are described in more detail below.

**Heterogeneity and *R_0_***

***Recipient dependence***

When three sub-populations are equally spaced in $\sigma/\iota$ trait space (*i.e.* tripartite isometric context, with a centroid fixed at $(\sigma= 0.5, \iota= 0.5)$), the covariation between susceptibility ($\sigma$) and infectiousness ($\iota$) is 0, regardless of the amount of heterogeneity present. Because the covariation of $\sigma$ and $\iota$ is 0, *R_0_* is the same in the homogeneous instance as all heterogeneous simulations (Figure S9).

Increasing heterogeneity in the tripartite non-isometric heterogeneity context leads to divergent results for *R_0_* (Figure S9C), as was seen in the bipartite heterogeneity context. When there is positive covariation between susceptibility ($\sigma$) and infectiousness ($\iota$), *R_0_* values increase; negative covariation reduces *R_0_* values (Figure S9C). Again, as for the bipartite heterogeneity case, the shape of the point cloud is constrained by how sub-populations can be positioned in $\sigma,\iota$ trait space.

***Donor dependence***

With equally spaced sub-populations (tripartite isometric heterogeneity), increasing heterogeneity in infectiousness increases *R_0_* values (Figure S9B). This is because *R_0_* values are determined by the maximum infectiousness ($\iota$) value. These results are broadly similar to the bipartite heterogeneity case.

Assuming tripartite non-isometric heterogeneity, the maximum infectiousness ($\iota$) value in each simulation determines the *R_0_* value, leading to a general increase in *R_0_* with increasing heterogeneity (Figure S9D). Though there is no change in *R_0_* if the maximum infectiousness ($\iota$) value does not change as heterogeneity increases (*i.e.* only an increase in heterogeneity in susceptibility ($\sigma$)). These findings are once again consistent with both the bipartite heterogeneity and the tripartite isometric heterogeneity contexts.

Figure S9. The effect of initial heterogeneity on *R_0_*. A) Results from the RD tripartite isometric heterogeneity context, where each point represents a unique simulation. The colour bar shows the covariance of susceptibility ($\sigma$) and infectiousness ($\iota$) in each simulation. B) Results from DD tripartite isometric heterogeneity, where each point represents a unique simulation. The colour bar shows the maximum infectiousness ($\iota$) value in each simulation. C) Results from RD tripartite non-isometric heterogeneity, where each point represents a unique simulation. The colour bar shows the covariance of susceptibility ($\sigma$) and infectiousness ($\iota$) in each simulation. D) Results from DD tripartite non-isometric heterogeneity, where each point represents a unique simulation. The colour bar shows the maximum infectiousness ($\iota$) value in each simulation. In all panels the dotted line shows where $R_{0}=1$.

**Host abundance**

In both the RD and DD tripartite isometric heterogeneity context, equilibrium host abundance increases with greater initial heterogeneity (Figure S10A & Figure S10B). Some total abundances increase by a much larger magnitude as heterogeneity values increase, while most increase by a smaller magnitude. While the DD tripartite isometric heterogeneity context (Figure S10B) is broadly similar to that of its RD counterpart, the lowest abundances are found at median levels of heterogeneity.

Similarly, the RD and DD tripartite non-isometric heterogeneity contexts show greater total host abundance with increasing heterogeneity (Figure S10C & Figure S10D). As with the bipartite heterogeneity abundance results (main text, Figure 4), simulations with higher *R_0_* values have lower total abundances, and simulations are grouped.

Figure S10. Effect of initial heterogeneity on total abundance of hosts at equilibrium. A) RD tripartite isometric heterogeneity context. B) DD tripartite isometric heterogeneity context. C) RD tripartite non-isometric heterogeneity context. D) DD tripartite non-isometric heterogeneity context.

**Change in heterogeneity**

Both the RD and DD tripartite non-isometric heterogeneity contexts broadly show the same pattern as for the bipartite heterogeneity context (compare Figure S11 with Figure S3). Although there is slightly more deviation from the $y=x$ line in both the RD tripartite non-isometric heterogeneity and the DD tripartite isometric heterogeneity context when compared to the RD tripartite isometric heterogeneity or the DD tripartite non-isometric heterogeneity cases, the general pattern shows that heterogeneity tends to be maintained at equilibrium (Figure S11).

Figure S11. Change in heterogeneity from the initial sub-population values to their equilibrium values. In each panel the dotted line shows $y=x$, each point represents at least one simulation, and darker points are multiple overlaid simulations. A) RD tripartite isometric heterogeneity context. B) DD tripartite isometric heterogeneity context. C) RD tripartite non-isometric heterogeneity context. D) DD tripartite non-isometric heterogeneity context.

**References**

Diekmann, O., Heesterbeek, J. & Roberts, M.G. (2010). The construction of next-generation matrices for compartmental epidemic models. *Journal of the Royal Society Interface*, 7, 873-885.

Laliberté, E. & Legendre, P. (2010). A distance‐based framework for measuring functional diversity from multiple traits. *Ecology*, 91, 299-305.
